# Clinical evaluation of non-invasive prenatal screening for the detection of fetal genome-wide copy number variants

**DOI:** 10.1186/s13023-022-02406-6

**Published:** 2022-07-08

**Authors:** Wenli Wang, Fengying Lu, Bin Zhang, Qin Zhou, Yingping Chen, Bin Yu

**Affiliations:** Changzhou Maternal and Child Health Care Hospital, No.16 Ding Xiang Road, Changzhou, 213003 Jiangsu China

**Keywords:** Non-invasive prenatal screening, Copy number variants, Chromosome microarray analysis, Prenatal diagnosis, Positive predictive value

## Abstract

**Objective:**

This study explores and discusses the possible factors affecting the positive predictive value (PPV) of non-invasive prenatal screening (NIPS) for the detection of fetal copy number variants (CNVs) in pregnant women.

**Methods:**

NIPS was performed for 50,972 pregnant women and 212 cases were suspected as fetal CNVs. Post additional genetic counseling for these women, 96 underwent invasive prenatal diagnosis (amniocentesis), following which they received chromosomal microarray analysis (CMA). We analyzed the PPV of NIPS for the detection of fetal CNVs and the possible interference factors that could affect the PPV.

**Results:**

Among the 96 pregnant women that received prenatal diagnosis by CMA, 37 cases were confirmed to be true positive for fetal CNVs with a PPV of 38.5%. There was no significant difference between the women with different NIPS indications. Five cases were reported as the false positive and false negative of fetal CNVs and the differences were mainly reflected in the inconsistency of chromosome fragments. Depending on the sizes of the CNVs, the PPVs were 48.7% for CNVs < 3 Mb, 41.4% for CNVs falling within 3 ~ 5 Mb, 42.9% for the CNVs falling within 5 ~ 10 Mb, and 14.3% for CNVs > 10 Mb. Based on the chromosomal locations of CNVs, the PPV(4.8%) of the chromosomes of group C(including chromosomes 6 ~ 12), was lower than that of the other groups (41.2% ~ 66.7%) (*p* = 0.021). However, there were no significant differences in the CNV characteristics, fetal fractions, unique reads, and the Z-scores between these groups.

**Conclusion:**

NIPS with a low-coverage sequencing depth has a certain effect on detection of fetal CNVs with the PPV of 38.5%. Chromosomal locations of CNVs may be the main factor that influences its effect. This study can contribute to an increased accuracy in genetic counseling and in predicting NIPS results that are positive for fetal CNVs.

## Introduction

Fetal microdeletion and microduplication syndromes (MMs) are caused by the presence of specific pathogenic copy number variants (CNVs) in the fetal genome. The most common chromosomal MMs, with an incidence rate of 1–1.7% [1], can cause serious clinical manifestations, including growth and development abnormalities, intellectual disability, and congenital malformations. Early detection and intervention is the most effective means to prevent fetal MMs. However, traditional prenatal screening and diagnosis methods are inefficient in detecting fetal MMs. At present, it mainly depends on invasive prenatal diagnosis, which greatly limits the effect of prenatal intervention. No other effective prevention and intervention methods have been developed yet. Therefore, more and more clinicians hope to have a more effective screening and diagnosis method in detecting fetal MMs.

Non-invasive prenatal screening (NIPS) is a globally well-established and effective method of prenatal screening. Initially, it was mainly performed to detect three common fetal aneuploidies, trisomy 21, trisomy 18, and trisomy 13 [2–4]. However, recently, studies have also reported its application in prenatal screening for detection of sex chromosome aneuploidies [3, 5, 6], fetal microdeletions/microduplications [7–9], and monogenic-inherited diseases [10–12].

In 2012, Jensen et al. [13] successfully extended the application of NIPS to examine fetal 22q11.2 microdeletion. Subsequently, other research groups have also proven the effectiveness of NIPS in detection of fetal MMs [14]. However, most of these studies focused on a limited number of common syndromes, such as 22q11.2 deletion, Prader-Willi, Angelman, 1p36 deletion, and cri-du-chat syndrome. The detection rates of these syndromes were approximately 90% in NIPS. Notably, many such studies were performed with high-coverage sequencing depth. Nevertheless, NIPS performed with a low-coverage sequencing depth might be an alternative method for screening fetal CNVs [15, 16]. But it still needs more clinical validation studies and technical improvement to achieve clinically acceptable accuracy [17].

Delayed clinical manifestations make it very difficult to identify MMs in neonates by routine follow-ups post prenatal screening and diagnosis. Thus, most studies evaluate only the positive predictive value (PPV) instead of the detection rate. According to recent reports, the PPV ranges from 11 to 80.56% [18]. It is believed that NIPS can detect the changes in fetal CNVs through cell-free fetal DNA, indicating the possibility of fetal MMs. However, the sample sizes used in previous studies were small, with some studies only including less than 10 cases of MMs. This limitation overlooks the variation in PPVs and the factors that may influence this value.

Here, the study focuses on detecting fetal CNVs by NIPS. We analyzed the NIPS data from 50,972 pregnant women and discussed the possible factors that influence the PPV. We hope that our study provides further insights into clinical prenatal genetic counseling in detecting fetal CNVs and improving the implications of NIPS.

## Materials and methods

### Ethics approval and consent to participate

The study design and protocol were reviewed and approved by the ethics committee of Changzhou Maternal and Child Health Care Hospital (No. 201501). All pregnant women received genetic counseling and gave informed consent before testing.

### Clinical subjects

From May 2012 to May 2021, 50,972 pregnant women underwent NIPS at Changzhou Maternal and Child Health Care Hospital. Calculation of AFP, free βHCG, free E3, maternal age and gestational age in the second trimester was used for prenatal serological screening [4]. The cases were categorized as such: women with advanced ages (13,452, 26.4%), high risk of serological screening (7492, 14.7%), intermediate risk of serological screening (11,291, 22.2%), voluntary demand (13,815, 27.1%), and others (4922, 9.7%), such as assisted reproductive conception and twins. The ages of these women ranged from 19 to 37 years old and the weeks of gestation ranged from 13 to 23^+5^. Post NIPS, 212 women were suspected to be positive for fetal CNVs and were called back for another round of genetic counseling. Invasive prenatal diagnosis (amniocentesis) was performed for 96 women, following which they underwent chromosomal microarray analysis (CMA).

### Non-invasive prenatal screening

In accordance with our previous reports [4, 19], massively parallel sequencing was performed on the Illumina NextSeq CN500 platform and analyzed by Bambni 2.0 software (Berry Genomics Co., Ltd). Fetal DNA concentration > 4% was the threshold for determining the quality of a sample. The sequencing depth was approximately 0.08X, and the threshold size for unique reads was ≥ 1.5 Mb. A Z-score > 3 defined an increase in copy number, whereas a Z score < − 3 defined a decrease in copy number.

### Prenatal diagnosis by CMA

Post amniocentesis, the women underwent prenatal diagnosis by CMA between 18–26 gestational weeks. This procedure has been described in our previous reports [20, 21]**.** Single nucleotide polymorphism array was processed using a commercial 750 K microarray chip (Affymetrix CytoScan 750 K Array). The data was analyzed using Chromosome Analysis Suite v3.2 software package. The public databases, DECIPHER, OMIM, ClinVar, ISCA, NCBI, and UCSC were used to interpret the data. The pathogenicities of identified CNVs were evaluated in accordance with the American College of Medical Genetics and Genomics guidelines [22].

### Statistical analysis

The data were analyzed by using EmpowerStats (X&Y solutions, inc.) and R software (http://www.R-project.org) [23]. The Chi-square test and F-test were used to compare differences in continuous variables between the groups. *p* < 0.05 was chosen to be statistically significant.

## Results

Among the 50,972 pregnant women that underwent NIPS in our prenatal diagnosis center, 212 women were suspected to have fetal CNVs. Post the second round of prenatal genetic consultation, 96 women consented to undergo prenatal diagnosis by CMA, while 116 declined. The rate of prenatal diagnosis was only 45.3%. Eventually, 37 women were confirmed as true positive for fetal CNVs with a PPV of 38.5% (Table 1). In accordance with the guidelines of American College of Medical Genetics and Genomics [22], we deciphered that 27 women (73.0%) out of 37 exhibited a pathogenic or likely pathogenic fetal CNVs, and these women all opted to terminate their pregnancies. On the other hand, 10 women (27.0%) exhibited fetal variants of unknown significance. Their children have not shown any obvious abnormalities after birth (Table 2). As shown in Table 1, the PPV of women with an intermediate risk of serological screening was the highest (60.0%), while that of women at advanced age was unexpectedly lower (30.8%). The women categorized in the assisted reproductive conception and/or twins groups had the lowest PPV (13.3%). However, there was no significant difference in PPV between the women with different NIPS indications.

**Table 1:** Maternal indications of fetal CNV detected by NIPS

Groups	*n*	Prenatal diagnosis by CMA			PPV (%)
		*n*	TP	FP	
Advanced age women	39	13	4	9	30.8
High risk of prenatal screening	31	12	4	8	33.3
Intermediate risk of prenatal screening	46	20	12	8	60.0
Voluntary demand	71	36	15	21	41.7
Others*	25	15	2	13	13.3
Total	212	96	37	59	38.5

*including assisted reproductive conception, twins, etc

**Table 2:** True positive fetal CNVs detected by NIPS

Case	NIPS					Prenatal diagnosis				Pregnancy outcome
	Result	Size (Mb)	Z-score	Fetal fraction	Unique reads (Mb)	Result	Size (Mb)	Syndrome	Type	
1	loss(1q21.1-q21.2)(144,500,000–147,499,999)	3.0	− 1.10	13.61	2.91	arr[GRCh37] 1q21.1q21.2(144,368,497–148,846,577) × 1	4.5	1q21.1 microdeletion syndrome	P	TOP
2	gain(1p36.32-p36.31)(4,000,000–6,499,999)	2.5	− 0.21	17.19	2.56	arr[GRCh37] 1p36.32p36.31(4,010,776–6,154,368) × 3	2.1		VUS	Birth
3	gain(2q13)(111,500,000–113,499,999)	2.0	1.07	7.69	2.35	arr[GRCh37] 2q13(110,980,107–113,132,395) × 3	2.1	2q13 recurrent region	P	TOP
4	loss(2q13)(111,500,000–113,499,999)	2.0	− 1.27	9.29	3.02	arr[GRCh37] 2q13(111,371,701–113,111,856) × 1	1.7	2q13 recurrent region	P	TOP
5	loss(2q13)(111,500,000–113,999,999)	2.5	− 0.74	10.47	3.86	arr[GRCh37] 2q13(111,371,701–113,111,856) × 1	1.7	2q13 recurrent region	P	TOP
6	gain(2p24.3)(12,500,000–16,499,999)	4.0	1.3	10.55	2.90	arr[GRCh37] 2p24.3(13,716,541–14,852,093) × 3	1.1	–	VUS	Birth
7	gain(2q13)(111,500,000–113,499,999)	2.0	1.0	4.85	2.65	arr[GRCh37] 2q13(110,973,853–113,111,856) × 3	2.1	2q13 recurrent region	P	TOP
8	gain(3p26.3-p26.2)(0–3,999,999)	4.0	1.98	12.54	3.25	arr[GRCh37]3p26.3p26.1(61,891–4,011,238) × 3	3.9	–	P	TOP
9	loss(3q29)(195,500,000–197,999,999)	2.5	− 0.79	11.64	3.29	arr[GRCh37] 3q29(195,718,751–197,340,833) × 1	1.6	3q29 microdeletion syndrome	P	TOP
10	gain(4p16.1-p15.33)(9,500,000–11,999,999)	2.5	− 0.30	21.04	1.97	arr[GRCh37]4p16.1(9,509,873–10,878,115) × 3	1.4	–	VUS	Birth
11	loss(4q35.1-q35.2)(185,000,000–190,499,999)	5.5	− 0.64	8.81	2.96	arr[GRCh37]4q35.1q35.2(185,630,915–190,098,342) × 1	4.5	–	P	TOP
12	gain(4q34.3-q35.2)(181,000,000–190,999,999)	10.0	2.64	25.03	3.32	arr[GRCh37]4q34.3q35.2(181,729,351–190,630,694) × 3	8.9	–	VUS	Birth
13	loss(4q35.2)(189,000,000–190,999,999)	2.0	− 0.17	11.22	3.93	arr[GRCh37]4q35.2(189,384,162–190,957,460) × 1	1.6	FSHD	P	TOP
14	gain(4q35.1-q35.2)(187,000,000–189,499,999)	2.5	− 0.97	13.72	2.23	arr[GRCh37]4q35.2(187,114,919–189,349,652) × 3	2.2	–	VUS	Birth
15	loss(5q23.1-q31.1)(115,500,000–134,999,999)	19.5	− 7.16	10.18	2.36	arr[GRCh37]5q23.1q23.3(115,614,571–130,478,768) × 1	14.7	–	P	TOP
16	gain(5p13.2-p13.1)(36,500,000–38,999,999)	2.5	1.34	22.91	2.50	arr[GRCh37]5p13.2p13.1(36,902,395–38,963,081) × 3	2.0	5p13 microduplication syndrome	P	TOP
17	loss(5p15.33-p14.1)(0–26,999,999)	27.0	− 3.98	21.07	3.11	arr[GRCh37]5p15.33p14.1(113,576–25,625,172) × 1	26.5	Cri du Chat syndrome	P	TOP
18	gain(8p23.1)(8,500,000–10,999,999)	2.5	0.96	19.6	2.27	arr[GRCh37]8p23.1(8,747,322–10,775,412) × 3	2.0	8p23.1 microduplication syndrome	P	TOP
19	gain(15q11.2-q12)(23,500,000–25,999,999)	2.50	1.99	16.65	2.22	arr[GRCh37]15q11.2(23,693,931–25,626,496) × 3	1.9	15q11-q13 microduplication syndrome	P	TOP
20	loss(16p13.11-p12.3)(15,500,000–17,999,999)	2.50	− 1.76	18.3	3.43	arr[GRCh37]16p13.11p12.3(15,338,152–18,172,468) × 1	2.8	–	P	TOP
21	gain(16p13.12-p13.11)(13,500,000–16,499,999)	3.0	0.86	5.79	2.22	arr[GRCh37] 16p13.11(14,900,042–16,278,133) × 3	1.4	–	LP	TOP
22	gain(17q12)(34,500,000–36,499,999)	2.0	1.31	12.39	3.46	arr[GRCh37] 17q12(34,440,088–36,311,009) × 3	1.9	17q12 microduplication syndrome	P	TOP
23	gain(17q12)(34,500,000–36,499,999)	2.0	0.38	11.1	4.79	arr[GRCh37]17q12(34,822,465–36,351,919) × 3	1.5	17q12 microduplication syndrome	P	TOP
24	gain(17p12-p11.2)(14,000,000–17,999,999)	4.0	0.36	12.61	3.02	arr[GRCh37] 17p12(14,087,918–15,441,802) × 3	1.4	CMT1A	P	TOP
25	gain(17p12)(13,500,000–15,999,999)	2.5	0.13	13.16	4.07	arr[GRCh37]17p12(14,087,918–15,428,902) × 3	1.3	CMT1A	P	TOP
26	gain(20p12.3-p12.2)(7,000,000–9,499,999)	2.0	1.34	9.50	2.82	arr[GRCh37]20p12.3(7,092,359–8,589,571) × 3	1.3	WPW	VUS	Birth
27	gain(22q11.21)(18,500,000–21,499,999)	3.0	0.84	8.57	3.76	arr[GRCh37] 22q11.21(18,970,561–21,800,471) × 3	2.8	22q11.2 microduplication syndrome	P	TOP
28	gain(22q11.21)(18,500,000–20,999,999)	2.5	0.31	8.44	3.00	arr[GRCh37]22q11.21(18,919,477–20,312,661) × 3	1.4	22q11.2 microduplication syndrome	P	TOP
29	gain(22q11.21)(18,500,000–21,499,999)	3.0	0.51	19.25	3.51	arr[GRCh37] 22q11.21(18,919,477–20,312,661) × 3	1.4	22q11.2 microduplication syndrome	P	TOP
30	gain(22q11.21)(18,500,000–21,499,999)	3.0	0.97	15.22	2.52	arr[GRCh37] 22q11.21(18,648,855–21,461,017) × 3	2.8	22q11.2 microduplication syndrome	P	TOP
31	gain(22q11.21)(18,500,000–21,499,999)	3.0	1.34	10.98	3.11	arr[GRCh37] 22q11.21(18,649,190–21,461,017) × 3	2.8	22q11.2 microduplication syndrome	P	TOP
32	gain(18q12.1-q23)(32,000,000–77,999,999)	43.5	7.19	11.04	2.19	arr[GRCh37]18q12.1(31,443,479–74,124,037) × 3	42.68	–	P	TOP
33	gain(13q21.31-q21.32)(63,000,000–66,999,999)	4.0	2.95	11.37	3.62	arr[GRCh37]13q21.31q21.32(62,944,040–66,680,852) × 3	3.7	–	VUS	Birth
34	gain(13q12.11-q12.13)(23,000,000–26,999,999)	4.0	0.54	15.57	2.90	arr[GRCh37] 13q12.12(23,473,289–24,958,572) × 3	1.5	–	VUS	Birth
35	gain(13q21.31-q21.32)(63,500,000–65,999,999)	2.5	2.79	7.04	2.4	arr[GRCh37] 13q21.31q21.32(63,855,596–65,903,526) × 4	2.0	–	VUS	Birth
36	loss(13q21.31-q21.32)(63,000,000–68,499,999)	5.5	− 4.30	15.38	3.92	arr[GRCh37]13q21.31q21.32(62,921,957–68,541,314) × 1	5.6	–	VUS	Birth
37	loss(13q12.3-q13.1)(29,000,000–32,499,999)	3.50	− 3.88	11.30	4.27	arr[GRCh37]13q12.3q13.1(29,195,848–32,460,071) × 1	3.3	–	P	TOP

*FSHD* Facioscapulohumeral muscular dystrophy, *CMT1A* Charcot-Marie-Tooth type 1A syndrome, *WPW* Wolff–Parkinson–White syndrome, *P* Pathogenic, *LP* Likely Pathogenic, *VUS* Variants of uncertain significance, *TOP* Termination of pregnancy, *Genome build* GRCh37

Moreover, Table 3 showed five cases with the discrepant results of fetal CNVs detected by NIPS and CMA. It was worth noting that the differences between both results were mainly reflected in the inconsistency of chromosome fragments. We conducted clinical treatment according to the results of prenatal CMA. Of which, case 5 and case 2 selected termination of pregnancy due to the pathogenic or likely pathogenic fetal CNVs. Other cases (case 1, 3 and 4) were confirmed as loss of heterozygosity or variants of uncertain significance, and they all obtained live births after continuing pregnancy. We are also closely observing the growth and development of these newborns.

**Table 3:** Discrepant results of fetal CNVs detected by NIPS and CMA

Case	NIPS					Prenatal diagnosis				Pregnancy outcome
	Result	Size (Mb)	Z-score	Fetal fraction	Unique reads (Mb)	Result	Size (Mb)	Syndrome	Type	
1	gain(2q14.1-q21.1)(114,500,000–130,999,999)	16.5	11.15	10.05	3.03	arr[GRCh37] 2q31.1q33.3(175,042,562–206,347,968)x2hmz	31.0	–	LOH	Birth
	gain(2q35-q37.3)(216,500,000–237,999,999)	21.5				arr[GRCh37] 2q11.1q13(95,550,957–114,045,382) × 2 hmz	18.5		LOH	
	gain(2p25.3-p24.1)(500,000–20,499,999)	20.0				arr[GRCh37] 2p15p11.2(62,680,101–87,053,152) × 2 hmz	24.4		LOH	
2	gain(5p15.33)(0–1,999,999)	2.0	0.71	13.97	3.86	arr[GRCh37] 5p13.2p13.1(36,902,395–38,963,081) × 3	2.0	5p13 microduplication syndrome	P	TOP
3	gain(9q33.3-q34.3)(129,000,000–140,499,999)	11.5	4.62	8.67	2.48	arr[GRCh37] 9q31.1q33.1(107,923,508–121,624,320) × 3	13.7	–	VUS	Birth
4	gain(16q23.1)(76,000,000–78,999,999)	3.0	4.18	10.23	2.16	arr[GRCh37] 16q23.1q24.3(78,969,980–90,146,366) × 2 hmz	11.0	–	LOH	Birth
						arr[GRCh37] 16p13.3p12.3(94,807–19,500,124) × 2 hmz	19.0		LOH	
5	gain(16q23.1)(76,500,000–78,499,999)	2.0	0.47	13.58	2.82	arr[GRCh37] 16p13.11(14,900,042–16,278,133) × 3	1.4	–	LP	TOP

*LOH* Loss of heterozygosity, *P* Pathogenic, *LP* Likely pathogenic, *VUS* Variants of uncertain significance, *TOP* Termination of pregnancy, *Genome build* GRCh37

**Fig. 1:**
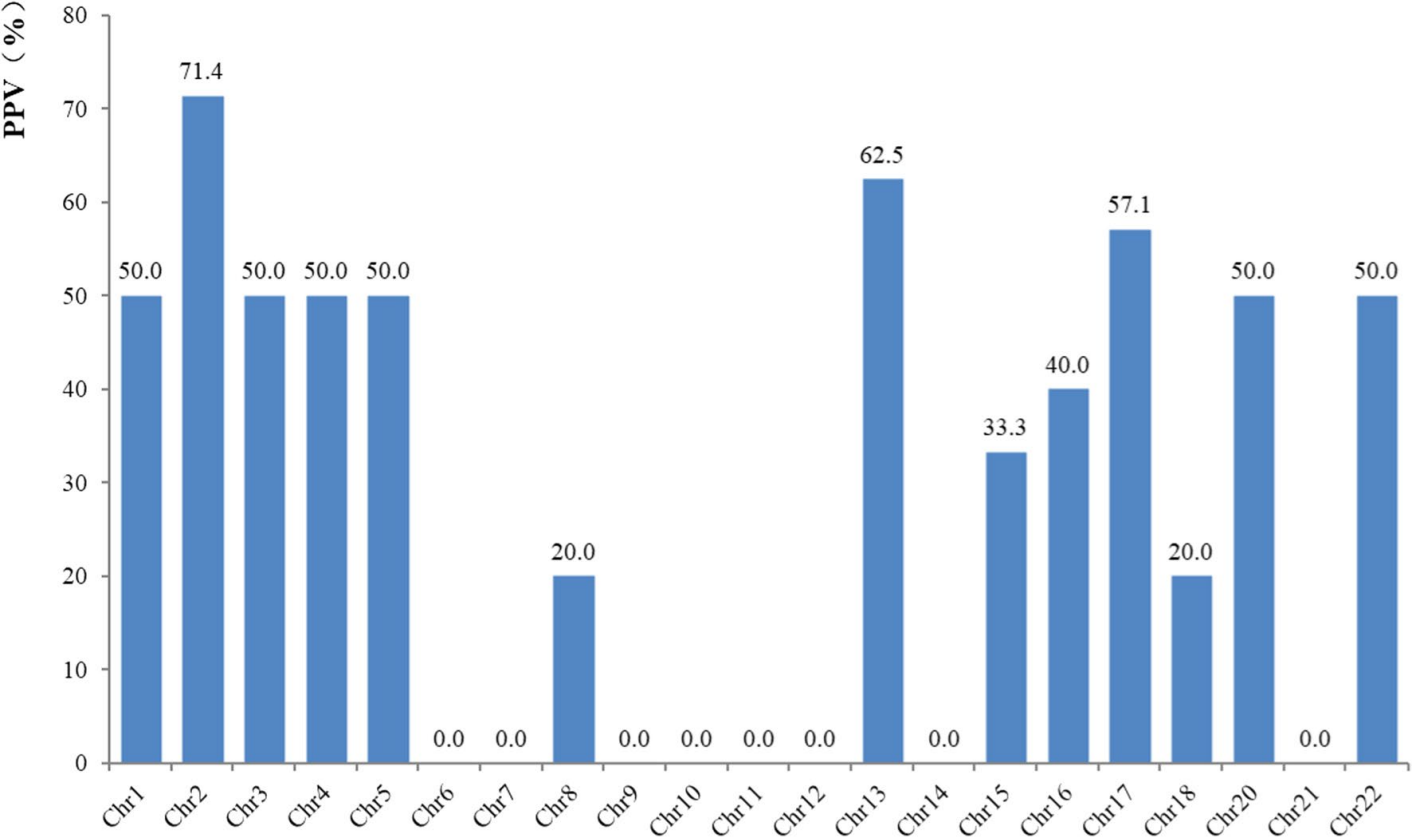
The PPV of NIPS for fetal CNVs in each Chromosome

A comparison between possible factors that could influence the PPV of NIPS were shown in Table 4. First, among the 96 women that tested positive for fetal CNVs, 69 (71.9%) were suspected to have segment gains and 27 (28.1%) to have segment losses. The PPV for these CNVs were 37.7% and 40.7% respectively; there was no significant difference (*p* = 0.782) between these PPVs. Second, the sizes of CNVs estimated by NIPS ranged from 2.0 to 43.5 Mb (median 3.0 Mb), whereas that verified by CMA ranged from 1.1 to 42.7 Mb (median 2.1 Mb). Moreover, the differences in the sizes of CNVs in 28 women (28/42, 66.7%) were less than 1 Mb, hinting at a consistent CNV size between two methods. Post comparing groups on the basis of the CNV sizes, the PPV was evaluated to be 48.7% for CNVs < 3 Mb, 41.4% for CNVs falling within 3 ~ 5 Mb, 42.9% for CNVs falling within 5 ~ 10 Mb, and 14.3% for CNVs > 10 Mb. Surprisingly, the PPV decreased with the increase in the CNV size; however, there was no significant difference in the PPVs observed between the groups (*p* = 0.170). Third, the PPV was marginally higher in women whose fetal fraction was > 10% than those whose fetal fraction was < 10% (44.4% vs. 27.3%, *p* = 0.101). Fourth, NIPS with low-coverage sequencing depth was performed in the present study. The average size of a unique read was 3.15 Mb. Additionally, there were no significant differences in the PPVs between the different groups on the basis of unique reads and the Z-score. Furthermore, upon comparing the PPV of different chromosome groups on the basis of the chromosomal location of CNVs, we found significant difference in the PPVs between the groups (*p* = 0.021). The PPV of most chromosome groups has a certain effect (4.2% ~ 60.0%). However, the PPVs for the chromosomes of group C (chr 6 ~ 12) were lower at only 4.8%. The relationship between chromosomal locations of CNVs and the detection efficiency of NIPS were shown in Table 5 and Fig. 1. Please note that the results of sex chromosome CNVs were not included in this study.

**Table 4:** Influencing factors of NIPS detection efficiency

Factors	Prenatal diagnosis by CMA			PPV (%)	*p* value
	*n*	TP	FP		
*CNVs characteristic*					
Segment gains	69	26	43	37.7	0.782
Segment losses	27	11	16	40.7	
*CNVs size*					
< 3 Mb	39	19	20	48.7	0.170
3 Mb ~ 5 Mb	29	12	17	41.4	
5 Mb ~ 10 Mb	7	3	4	42.9	
> 10 Mb	21	3	18	14.3	
*Fetal fraction*					
< 10%	33	9	24	27.3	0.101
≥ 10%	63	28	35	44.4	
*Unique reads*					
< 3 Mb	52	18	34	34.6	0.762
3 Mb ~ 4 Mb	36	15	21	41.7	
> 4 Mb	8	4	4	50.0	
*CNVs Z-score*					
Within 3	68	32	36	47.1	0.007
Beyond 3	28	5	23	17.9	
*Chromosome grouping*					
A group (chr 1,2,3)	15	9	6	60.0	0.021
B group (chr 4,5)	16	8	8	50.0	
C group (chr 6,7,8,9,10,11,12)	21	1	20	4.8	
D group (chr 13,14,15)	14	6	8	42.9	
E group (chr 16,17,18)	17	7	10	41.2	
F group (chr 19,20)	2	1	1	50.0	
G group (chr 21,22)	11	5	6	45.5

**Table 5:** Chromosome location and NIPS detection efficiency

Chromosome Numbers	*n*	Prenatal diagnosis by CMA			PPV (%)
		*n*	TP	FP	
Chr1	7	4	2	2	50.0
Chr2	11	7	5	2	71.4
Chr3	10	4	2	2	50.0
Chr4	13	10	5	5	50.0
Chr5	7	6	3	3	50.0
Chr6	2	2	0	2	0.0
Chr7	38	3	0	3	0.0
Chr8	14	5	1	4	20.0
Chr9	9	6	0	6	0.0
Chr10	6	0	0	0	0.0
Chr11	4	2	0	2	0.0
Chr12	6	3	0	3	0.0
Chr13	8	8	5	3	62.5
Chr14	12	3	0	3	0.0
Chr15	5	3	1	2	33.3
Chr16	12	5	2	3	40.0
Chr17	11	7	4	3	57.1
Chr18	8	5	1	4	20.0
Chr20	10	2	1	1	50.0
Chr21	3	1	0	1	0.0
Chr22	16	10	5	5	50.0
Total	212	96	37	59	38.5

## Discussion

Clinical application of NIPS in prenatal screening for fetal CNVs is gaining increasing momentum. However, there are many problems yet to be solved. For example, these questions are yet unanswered: How to evaluate the effect of NIPS scientifically? How to reduce the factors interfering with NIPS efficiency and improve the accuracy of NIPS? In the present study, we evaluated the PPV of NIPS from a large sample size cohort, and reached a conclusion that the PPV of NIPS with low-coverage sequencing depth for detection of fetal CNVs was 38.5%. Furthermore, we evaluated several factors that could affect the PPV and found that it was closely related to the chromosomal locations of CNVs.

It is well known that microdeletion/microduplication syndromes exhibit great variation and complicated clinical manifestations. Despite routine follow-ups after prenatal screening, it is very difficult to identify MMs in neonates. Most studies have used the PPV to evaluate the screening effect of NIPS. Only a few reports have assessed the detection rate of some varieties of MMs. Our study reported that the PPV for NIPS with low-coverage sequencing depth for detection of fetal CNVs (38.5%) was higher than that reported in similar studies, such as those reported by Yang (30.96%) [24], Hu (36.11%) [16] and Chen (28.99%) [15]. Recently, it has been reported that the PPV could improve with high-coverage sequencing. This is evident in the findings of Yang’s group upon comparing the PPV of two NIPS data with different sequencing depths, where they found that the PPV of NIPS Plus (0.4X) was 12.65% higher than that of NIPS (0.15X) (43.61% vs. 30.96%) [24]. Shi et al. also reported that the PPV of NIPS Plus for detection of MMs with unremarkable ultrasound findings was 50% [18]. However, these findings did not significantly improve the PPV when compared to our results. NIPS based on low-coverage sequencing depth has a certain effect on prenatal screening for detection of fetal CNVs too. It is undeniable that the detection effect of NIPS for fetal CNVs is not satisfactory and much worse than that of fetal chromosome aneuploidy [25]. Some study reported that combining with maternal age, prenatal serological screening and/or ultrasound scanning could improved NIPS screening performance [26]. Fetal MMs has become a great challenge for prenatal screening and diagnosis. Traditional prenatal screening and diagnosis seemed to no good effective for fetal MMs. Therefore, at present, NIPS may be a more feasible method for clinical prenatal screening of fetal CNVs.

Few studies have focused on the factors influencing the efficiency of NIPS. One such factor is the CNV size, wherein the sensitivity of NIPS enhanced with increase in CNV size in some common MMs [9, 27]. For example, the sensitivity for detecting CNVs > 10 Mb was higher (91.67%) than for CNVs < 5 Mb (68.42%). Ye et al. [17] also reported the poor sensitivity of NIPS in CNVs < 2 Mb by a retrospective study. However, the PPV didn’t exhibit such a trend [28]. Our findings concurred with these observations as we also did not observe a significant difference in the PPVs for CNV sizes between groups. Surprisingly, we did observe that the larger the CNV size, the lower the PPV. While some studies have reported that the PPV of CNVs > 10 Mb is the lowest [15, 24], we could not obtain strong evidence to explain this strange problem. We conjecture that this may be attributed to the interference of chromosomal location of CNVs with the PPV estimation. Among the 16 false positive cases with CNVs > 10 Mb in the present study, 50% were because of MMs on chromosomes 7, 9, and 14. In the present study, we found that CNV characteristics, fetal fractions, unique reads, and the Z-scores had no significant influence on the PPV. However, more studies investigating this aspect are needed as the current literature is limited.

There are some limitations to this study: the sample size was not large enough. The rate of prenatal diagnosis was low, only 96 women received prenatal diagnosis and were included in the later analyses. No in-depth investigation could be performed to determine additional influencing factors, and we did not analyze the sex chromosome CNVs.

In conclusion, NIPS performed with low-coverage sequencing depth has a certain effect on prenatal screening for detection of fetal CNVs and has a PPV of 38.5%. The chromosomal location of CNVs may be the main influencing factor governing the PPVs. We believe that our findings can contribute towards increasing the accuracy in prediction and genetic counseling when dealing with cases positive for fetal CNVs as detected by NIPS.

## Data Availability

The datasets presented in this article are not readily available because Regulations on the management of human genetic resources in China. Requests to access the datasets should be directed to the corresponding author.
